# Spillway-Induced Salmon Head Injury Triggers the Generation of Brain αII-Spectrin Breakdown Product Biomarkers Similar to Mammalian Traumatic Brain Injury

**DOI:** 10.1371/journal.pone.0004491

**Published:** 2009-02-13

**Authors:** Ann Miracle, Nancy D. Denslow, Kevin J. Kroll, Ming Cheng Liu, Kevin K. W. Wang

**Affiliations:** 1 Environmental Sustainability Division, Pacific Northwest National Laboratory, Richland, Washington, United States of America; 2 Department of Physiological Sciences and Center for Environmental and Human Toxicology, University of Florida, Gainesville, Florida, United States of America; 3 Center of Innovative Research, Banyan Biomarkers Inc, Alachua, Florida, United States of America; 4 Center for Neuroproteomics and Biomarkers Research, Department of Psychiatry, McKnight Brain Institute, University of Florida, Gainesville, Florida, United States of America; University of Lethbridge, Canada

## Abstract

Recent advances in biomedical research have resulted in the development of specific biomarkers for diagnostic testing of disease condition or physiological risk. Of specific interest are αII-spectrin breakdown products (SBDPs), which are produced by proteolytic events in traumatic brain injury and have been used as biomarkers to predict the severity of injury in humans and other mammalian brain injury models. This study describes and demonstrates the successful use of antibody-based mammalian SBDP biomarkers to detect head injury in migrating juvenile Chinook salmon (*Oncorhynchus tshawytscha*) that have been injured during passage through high-energy hydraulic environments present in spillways under different operational configurations. Mortality and injury assessment techniques currently measure only near-term direct mortality and easily observable acute injury. Injury-based biomarkers may serve as a quantitative indicator of subacute physical injury and recovery, and aid hydropower operators in evaluation of safest passage configuration and operation actions for migrating juvenile salmonids. We describe a novel application of SBDP biomarkers for head injury for migrating salmon. To our knowledge, this is the first documented cross-over use of a human molecular biomarker in a wildlife and operational risk management scenario.

## Introduction

The migration of juvenile salmonids downriver on the Columbia and Snake Rivers has been contested to be compromised due to the multiple hydropower facilities located on these rivers. Physical injury resulting from impacts with spillway structures and turbines and hydraulic forces associated with spill and sudden depth changes are two main hazards associated with hydropower-related passage. Laboratory studies of the effect of exposure to severe hydraulic events on juvenile salmonids have found a variety of adverse effects caused by strike, shear, pressure gradients, and disorientation. Recent studies have also found that fish exposed to high shear and turbulence are subject to direct injury and are more susceptible to predation than migrating fish which have non-turbulent passage [1]. Current efforts to assess these and strike-related injuries are performed using a direct injury and mortality approach by gross observation up to 48 hours post passage or condition treatment. Subacute injuries are not routinely measured as there is no available metric to determine non-visible injuries short of assessments for disorientation following laboratory treatment, and this type of observation is not used in field studies for testing hydropower structure configurations. Injury-based biomarkers may serve as quantitative indicators of injury severity. Because head injury likely results from physical trauma, such as impacting a physical structure or extreme high velocities, the development of a biomarker assay to quickly assess subacute physical injury and recovery is essential to determine the impact of hydropower structures on fish health.

Recent advances in biomedical research have resulted in the development of a specific mammalian biomarker to rapidly assess traumatic brain injury [2]–[6]. Breakdown products of the cytoskeletal protein αII spectrin (280 kDa) are produced following either calpain and/or caspase proteolysis; each digestion giving rise to different sized spectrin breakdown products (SBDPs) [7]–[9]. Because rapid treatment following suspected traumatic brain injury (TBI) is linked with survival and recovery, having a biomarker that can be used as a diagnostic has great human health importance [10].

Molecular biomarkers used in human health applications are often applicable to other mammals; specifically for veterinary medicine [11], [12]. However, application for risk management of non-mammalian wildlife has been limited or difficult due to the lack of specific biomarkers for exposure and effect to environmental, chemical, or physical stressors [13]–[16]. This study details the unique application of a molecular biomarker for human brain injury in a non-mammalian species for use in wildlife risk management.

## Materials and Methods

### Fish collection

Chinook salmon juvenile fish (80–110 mm total length) used for antibody cross-reactivity experiments were raised at the Pacific Northwest National Laboratory Aquatics Research Facility in Richland, WA, following transport from Lyons Ferry Hatchery (Starbuck, WA) according to facility guidelines based US Fish and Wildlife Service fish hatchery management practices, and in accordance with approved animal care and use protocols. These fish were used as a source of brain tissues for protease treatments and were not subjected to any injury-inducing conditions, and are referred to as hatchery control fish. Juvenile fish used in field testing originated from a single cohort (110–140 mm total length) from Lyons Ferry Hatchery and were maintained prior to and after passage testing in 6 foot circular tanks with constant Snake River water flow.

### Field treatments and Tissue Dissection

Field treatments involved the use of balloon-tagged hatchery fish into pipes constructed to release fish at predefined elevations through various spillways as part of a US Army Corps of Engineers contracted study at Ice Harbor Lock and Dam, WA, in the spring of 2006. The study's objective was to assess direct injury and mortality as the result of passage under varying operating conditions involving flow regimes [see 17], [18 for description of similar studies]. Subsets of fish were collected from different passage conditions according to facility guidelines based on US Fish and Wildlife Service fish hatchery management practices, and in accordance with approved animal care and use protocols. A control group consisted of a subset of the fish obtained for the study that were handled similarly to the passage treatments, but were released through the juvenile fish bypass facility chute at Ice Harbor Lock and Dam, and did not experience spillway passage hydraulic conditions or deflector obstructions. The control group in the field tests essentially served as a sham group to assess impacts from spillway conditions and not from tagging and handling conditions. Bypass chute conditions essentially consisted of a horizontal raceway flume with a 2 foot drop from discharge to collection. Fish were collected post-passage, examined for visible external injury (eye hemorrhage, eye loss, descaling, and abrasions) and sampled for brain tissue following the conclusion of the standard 48-hour holding time for direct mortality (i.e., balloon-tag) observations. Twenty fish each from different passage scenarios and 10 control fish were collected and euthanized in 250 mg/L tricaine methane sulfonate followed by decapitation prior to excising whole brain tissue. Brains were removed, placed in individual cryotubes, and flash-frozen in liquid nitrogen. Samples were stored at −80°C.

### Salmon brain lysate preparation and protease treatment

The brain samples were pulverized with a small mortar and pestle set over dry ice to a fine powder. The pulverized brain tissue powder was then lysed for 90 min at 4°C with 50 mM Tris (pH 7.4), 5 mM EDTA, 1% (v/v) Triton X-100, 1 mM DTT, 1× protease inhibitor cocktail (Roche Biochemicals). The brain lysates were then centrifuged at 8,000×g for 5 min at 4°C to clear and remove insoluble debris, snap-frozen and stored at −80°C until used. Protein concentrations of tissue lysates were determined by DC Protein assay (BioRad) with albumin standards. For *in vitro* protease digestion, freshly prepared normal rat or hatchery control salmon brain lysates (50 ug) were treated with purified proteases (human porcine calpain-2 (Calbiochem) or recombinant human caspase-3) with a substrate to protease ratio of 1/200 to 1/40 in a buffer containing 50 mM HEPES (pH 7.4), 10 mM dithiothreitol, 1 mM EDTA (for caspase-3) or 2 mM excess CaCl_2_ (for calpain-2) for 30–60 min at room temperature. For activating endogenous proteases in salmon brain, freshly prepared normal salmon brain lysate (50 ug) was incubated in a buffer containing 2 mM CaCl_2_, 50 mM HEPES (pH 7.4), 10 mM dithiothreitol, (for calpain activation), or in a buffer containing 10 mM dATP, 10 µM cytochrome C, 50 mM HEPES (pH 7.4), 10 mM dithiothreitol, 1 mM EDTA (for capase activation) for 2 h at room temperature.

### SDS-polyacrylamide gel electrophoresis and electrotransfer

Protein samples were prepared for sodium dodecyl sulfate-polyacrylamide gel electrophoresis (SDS-PAGE) in two-fold loading buffer containing 0.25 M Tris (pH 6.8), 0.2 M DTT, 8% SDS, 0.02% bromophenol blue, and 20% glycerol in distilled H_2_O. Twenty micrograms (20 µg) of protein per lane were resolved by SDS-PAGE on 6.5% Tris/glycine gels for 2 h at 200 V. Following electrophoresis, separated proteins were laterally transferred to polyvinylidene fluoride (PVDF) membranes in a transfer buffer containing 0.5 M glycine, 0.025 M Tris-HCl (pH 8.3), and 10% methanol at a constant voltage of 20 V for 2 h at 4°C in a semi-dry transfer unit (Bio-Rad).

### Immunoblotting and densitometry analysis

After electrotransfer, membranes were blocked for 1 h at ambient temperature in 5% non-fat milk in TBS and 0.05% Tween-2 (TBST), then incubated in primary antibody (αII-spectrin monoclonal antibody, Affinity Res. Prod. Nottingham, UK #FG6090 that is further biotinylated [8] or rabbit anti-SBDP120 [19] in TBST with 5% milk at 1/1,000 to 1/3,000 dilution as recommended by the manufacturer) at 4°C overnight, followed by four washes with TBST and a 2-hour incubation at ambient temperature with either a secondary antibody linked to horseradish peroxidase (enhanced chemiluminescence, (ECL) method), or biotinylated secondary antibody (Amersham), followed by a 30 min incubation with strepavidin-conjugated alkaline phosphatase (colorimetric method). Colorimetric development was performed with a one-step BCIP-NBT reagent (Sigma). Molecular weights of intact proteins and their potential αII-spectrin breakdown products (SBDPs) were assessed using rainbow colored molecular weight standards (GE Health Tech.). Semi-quantitative evaluation of protein and SBDP levels were evaluated via computer-assisted densitometric scanning (Epson XL3500 high resolution flatbed scanner) and image analysis with Image J software (version 1.6) (NIH). Regression analyses were performed using SigmaPlot 10.0 (Systat).

## Results

### Conservation of SBDPs in salmon brain

**Figure 1:**
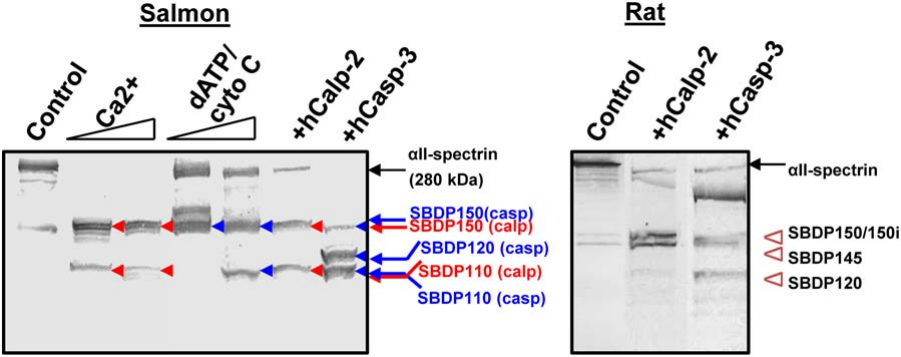
Endogenous and exogenous calpain and caspase-3 digestion of αII-spectrin in salmon brain lysate. Control Salmon brain was either untreated, or incubated with dATP and cytochrome-C (to activate endogenous caspase) or with CaCl_2_ (to activate endogenous calpain) or digested with exogenously added human calpain-2 or caspase-3. The masses of SBDP fragments or various molecular weight markers are as indicated (Left panel). Fragments produced by calpain are indicated with red arrows (SBDP150 and SBDP110), while those produced by caspase are indicated with blue arrows (SBDP150, SBDP120 and SBDP110). For comparison, rat brain lysate (control, or digested with calpain-2 (producing SBDP150 and SBDP145) or caspase-3 (producing SBDP150i and SBDP120) was also included (right panel), as described before [8], [27], [28].

Whole salmon brain homogenates showed some similar SBDPs to rat SBDPs when digested with calpain and caspase and detected via Western blot with biotinylated anti-mammalian αII-spectrin (**Fig. 1**). As controls, rat brain lysates (untreated or digested with human calpain-2 or caspase-3) were also included. Calpain-digestion of rat brain lysates produced the known SBDPs of 150 kDa and 145 kDa (SBDP150, SBDP145), while capase-3 produced SBDPs of 150 kDa and 120 kDa (SBDP150i, SBDP120) [7]–[9].

In the case of the salmon brain lysate, we observed some of the same fragment sizes and some that were different. Digestion with either exogenously added human caspase-3 or activation of the endogenous salmon caspase with dATP and cytochrome C, resulted in the production of a 110 kDa (SBDP110) not seen in the rat samples. In addition the exogenous caspase-3 produced a 120 kDa (SBDP120) in salmon brain lysate that is normally seen in the rat. Both enzymes produced the 150 kDa (SBDP150) in the fish brain. These observations suggest that αII-spectrin of salmon contains several of the preferred cleavage sites for mammalian caspase-3 to produce SBDP120 and SBDP150, but that it has additional sites that are sensitive to both the mammalian and endogenous caspase enzymes.

In the case of calpain, both the exogenously added enzyme and the endogenous enzyme (activated by the addition of CaCl_2_) produced a fragment of 150 kDa, as seen in the rat. But, both enzymes also produced a SBDP of 110 kDa, which again was not seen in the rat and did not produce the 145 kDa SBDP as seen in the rat controls (**Fig. 1**). Regardless, the anti-mammalian αII-spectrin monoclonal antibody was demonstrated to cross-react with salmon αII-spectrin and its various SBDPs. Although a basal level of SBDPs were observed in the control fish depicted in Figure 1, intact αII-spectrin is the most abundant protein detected, and is representative of the expression profile of a non-injured fish.

**Figure 2:**
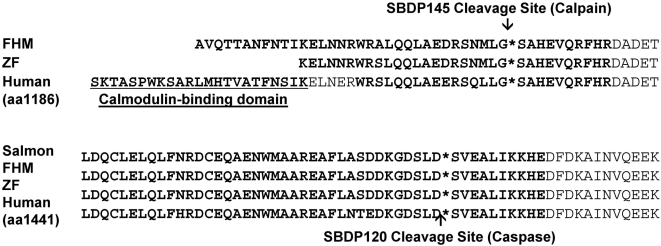
αII-spectrin partial sequence alignment with human and fish counterparts. Reference amino acid number is given for human sequence. Shown are regions pertinent to calpain proteolysis and caspase proteolysis with available salmon (CA049982), zebrafish (ZF, NP001091958), fathead minnow (FHM, DT285007) and human αII-spectrin (AAB41498) amino acid sequences are compared.

Amino acid alignment of a partial salmon predicted amino acid sequence with two known fish protein sequences; zebrafish [20] and fathead minnow, and the human protein sequence [21] show conservation at the SBDP120 cleavage site, which was one of the observed products following caspase cleavage (**Fig. 2**). The cleavage site for calpain proteolysis (SBDP145) appears to be conserved in both fathead minnow and zebrafish, but cannot be confirmed for salmon, at the present time.

### Field Testing

**Figure 3:**
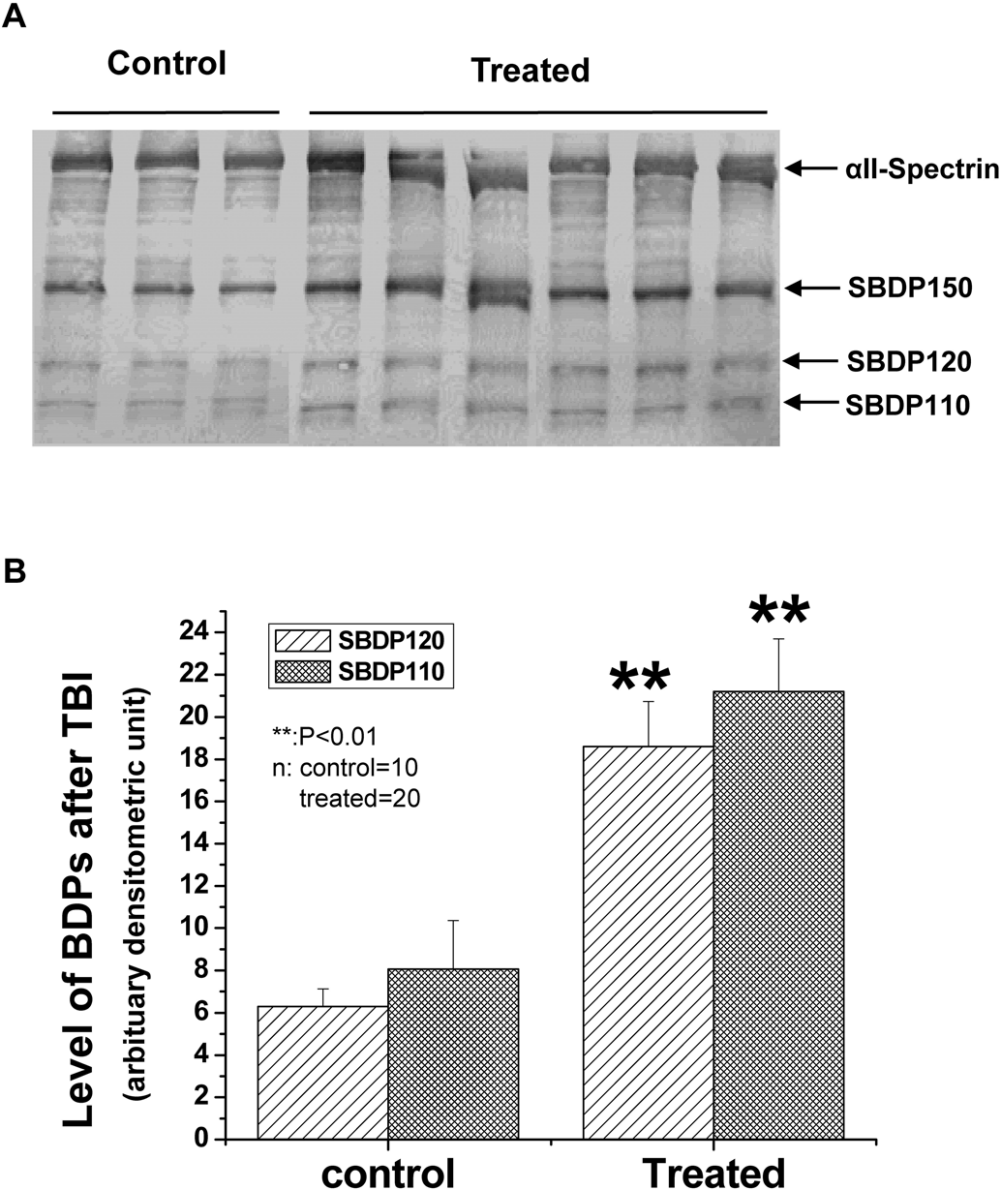
αII-spectrin proteolysis in salmon brain following spillway force induced brain injury. (A). αII-spectrin (280 kDa) and its fragments SBDP150, SBDP120 and SBDP110 are as indicated. Representative samples are shown. (B). Quantification of SBDP120 and SBDP110 elevation in injured salmon brain vs. controls. A total of n = 10 for normal and n = 20 for injured were quantified. Statistical significance (P<0.01, Student T-test) for both SBDP120 and SBDP110 are indicated with **.

Ten control and twenty passage treatment fish were assessed for expression of SBDPs using biotinylated anti-mammalian αII-spectrin (**Fig. 3A**). Densitometric analyses of differentially expressed SBDPs found significant differences between the control and passage treated groups with *p*<0.01 for SBDP120 and *p*<0.01 for SBDP110 (**Fig. 3B**). In both cases, the SBDP120 and SBDP110 levels increased about 2-fold after salmon brain injury (**Figure 3B**). We also note that there is a consistent basal level of SBDPs even in control brain, representing turnover of αII-spectrin in salmon brain tissues.

**Figure 4:**
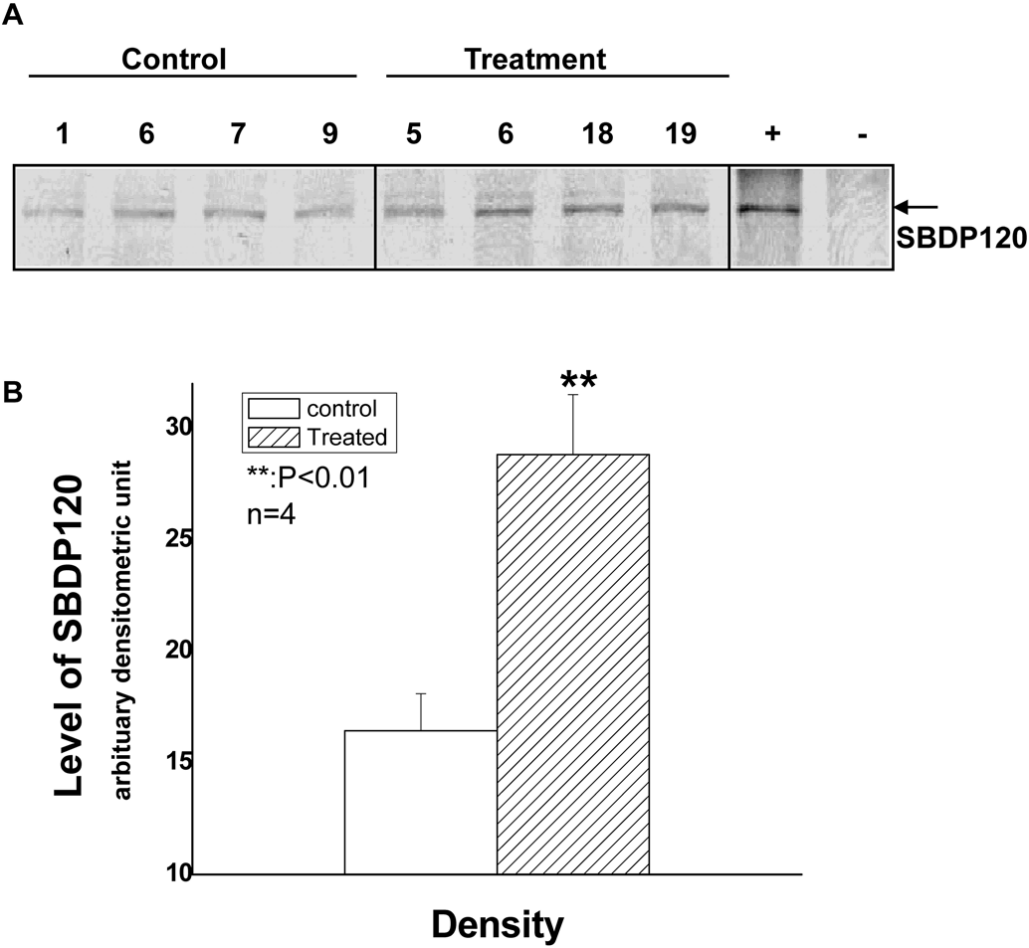
SBDP120-specific antibody confirming the presence of the SBDP120 fragment in injured salmon brain. Rabbit antibody developed specifically to detect mammalian SBDP120 fragment [19] was used in Western blot analysis of salmon brain lysate for controls and injured (treatment) salmon. (A) representative blot of salmon brain samples (4 controls and 4 injured) are shown. Positive and negative controls were rat brain lysate digested with caspase-3 and calpain-2, respectively (right two lanes). (B) A total of n = 8 from each group were used for densitometric analysis of the SBDP120-specific band intensity, achieving statistical significance (**, P<0.02, Student T-test) between control and injured groups.

We further confirm that the SBDP120 observed is identical to the SBDP120 observed in mammalian brain following brain injury by showing that an antibody developed specifically to detect the SBDP120 fragment in mammalian systems [19]. Using a subset of 8 samples per group (which was dictated by availability of brain lysates), the SBDP120 fragment was detected in injured salmon brain lysates (**Fig. 4**). The results further confirmed that there is a statistically significant two-fold elevation (p<0.02) of caspase-generated SBDP120 in the injured salmon brains (treatment group) when compared to the control brain group (**Fig. 4b**). As for SBDP110, at the present time, we cannot confirm whether it was generated by endogenous calpain or caspase in salmon brain, since both proteases appear to generate an SBDP of 110 kDa (see **Fig. 2**) when chemically activated and no SBDP110 fragment-specific antibody tools are available.

**Figure 5:**
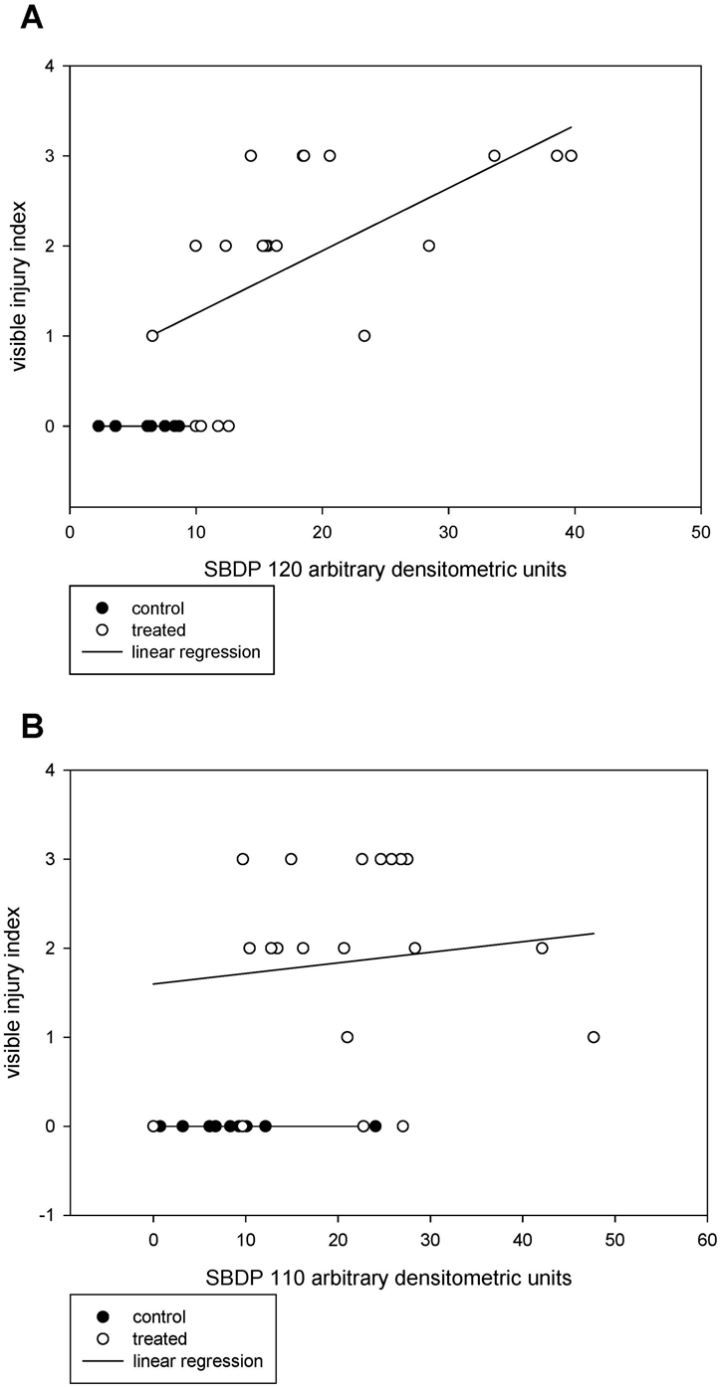
Correlation between SBDP expression with visible injury index. Open circles represent individual passage treatment fish (n = 20), while closed circles indicate individual control fish (n = 10). Linear regression lines are depicted for each group. A. SBDP120 linear regression r^2^ = 0.34. B. SBDP110 linear regression r^2^ = 0.01.

Numerical injury index scores (1–3) were given to injuries to reflect injury severity with increasing physical trauma (abrasion/descaling<eye hemorrhage<eye loss). When compared to observable injuries, increasing expression of SBDP120 showed correlation above a relative expression level of 10 (densitometric units), but expression was also seen above control level without obvious injury (**Fig. 5A**, r^2^ = 0.34). The pattern is less clear for increasing expression of SBDP110 (**Fig. 5B**, r^2^ = 0.01). A few of the treated fish and one control fish that showed no visible injury had relatively high levels of SBDP110 expression. With the exception of a few treatment fish, most treatment fish had expression levels of SBDP greater than 10.

## Discussion

The routine application of molecular biomarkers in field monitoring and testing has been limited to veterinary health. However, increasing use of risk-surveillance approaches for identification of health issues in livestock are being accepted for both strategic and operational purposes [22]. While disease and vector transmission implications to human health are the drivers for this type of risk management, the animal populations in question are monitored for disease exposure, overall health and productivity. The extension of incorporating molecular markers for contaminant/disease exposure has been documented for assessing wild populations of organisms at all trophic levels [16], [23], [24]. However, the specificity of many of these markers, or biomarkers, remains questionable for wildlife management practices [25], [26]. Aside from biological or chemical exposures, there has been no documented assessment of acute or sub-lethal injury impacts resulting from physical trauma in wildlife. The importance of this study illustrates the success in using a biomarker for mammalian or human head injury to determine head injury in migrating juvenile salmon as a result of hydropower passage management; and represents the first study to examine the efficacy of a human-based molecular biomarker metric for risk management.

Unique and differential αII-spectrin breakdown products were first shown to be generated by calpain and caspase proteases in cell culture neurotoxic models and *in vitro*, producing SBDP150/SBDP145 and SBDP150i/SBDP120, respectively [27], [28]. Pike and colleagues were also the first to observe the same major SBDPs in different affected rat brain regions [9] and in the cerebrospinal fluid (CSF) compartment [8] following experimental traumatic brain injury (controlled cortical impact). In the current study, spillway force-induced brain injury in salmon brain robustly produces SBDPs parallel to those produced by their mammalian counterparts (**Fig. 3** **,** **4**). When compared with the visible injury observations 48 hours post passage, the trends in passage impact correlate with the presence of SBDP120. In particular, the abundance of SBDP120 shows increasing expression with increasing visible injury. Predicted amino acid sequence homology for the SBDP120 cleavage site, and binding of the specific SBDP120 antibody provide strong evidence for conservation of functional homology. Based on the known mammalian expression pattern, the increase in expression is hypothesized to be evidence of apoptotic breakdown of intact αII-spectrin, a likely event that would occur following physical trauma to brain tissues. The lack of complete homology of mammalian SBDPs in salmon brain tissues may be related more to the evidence of genome duplication in the salmonids [29] and the loss of function due to redundancy [30], or adaptation for different functions. Without a complete αII-spectrin sequence in salmon, these remain assumptions.

**Figure 6:**
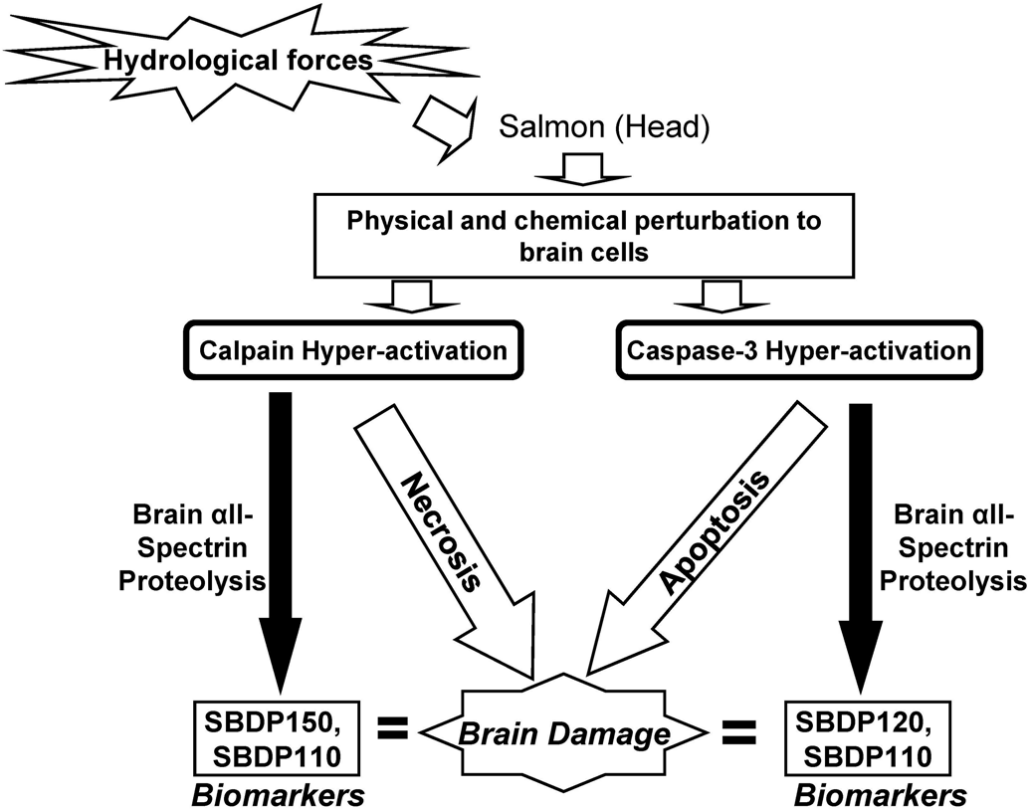
Schematic for protease-mediated salmon brain injury and the production of SBDP biomarkers in the brain. For details, see Discussion.

Two different pathways that lead to brain cell death are depicted in **Figure 6** **,** showing how calpain and caspase proteases may break down salmonid brain cellular proteins during both acute necrosis and delayed apoptosis phases of salmon head injury, respectively. Therefore, αII-spectrin was degraded by these two proteases, producing SBDPs as possible spillway force-mediated salmon brain injury biomarkers. Based on this study, there appears to be basal levels of SBDPs expressed in salmon brain tissues (**Fig. 3**), which may indicate a normal turnover of neuronal cells experienced during juvenile growth. Indeed, Zupanc [31] reviews the capability of teleost brain tissues to display continuous neurogenesis postembryonically and into adulthood. Soutschek and Zupanc [32], [33] describe the neuronal turnover process as apoptotic regulation, but increased apoptosis is also seen in response to injury and removal of injured or affected cells [reviewed in 31]. Although the difference in hydraulic force is orders of magnitude lower than passage through the spillway structures, it is possible that the bypassed fish used as a field control experienced some mild injury, and may account for the presence of SBDPs. However, the hatchery control fish used for the protease digests did not experience any field-related hydraulic forces, and the presence of SBDPs in the control fish lends support to the neurogenesis hypothesis. The increase in SBDP120 appears to correlate with observations of head injury and could be used as a potential biomarker for subacute brain damage induced by migration passage. These results have increased significance following the recent Biological Opinion for the Federal Columbia River Power System [34] for cooperating agencies to develop configuration and operations plans that detail overall survival improvements for in-river migrating fish.

Although currently there is no threshold head injury biomarker expression level known that would indicate an outcome such as recovery, impaired health, or delayed mortality, the results can be compared among passage types to determine optimal passage trajectories, and are an improvement over current assessments that involve visible injury assessment for hundreds of fish per passage type. Non-lethal sampling is a preferred alternative to sampling brain tissue, and cerebral spinal fluid (CSF) is the biofluid used for human biomarker analysis [5], [6], [8]. However, CSF is not a viable alternative for juvenile fish that range in total size from 80–140 mm. Blood samples (including serum or plasma) may be a preferable biofluid for salmon brain injury biomarker monitoring. We are working to get salmon specific antibodies to enable the detection of the biomarker in blood. In addition, future work to assess degree of injury in relation to specific outcomes are underway and will include temporal studies under defined, laboratory simulations to determine more specific biomarker metrics for longer-term prognosis of fish outcome.
